# KCNG4 Genetic Variant Linked to Migraine Prevents Expression of KCNB1

**DOI:** 10.3390/ijms25168960

**Published:** 2024-08-17

**Authors:** Gabriel Lacroix, Shreyas Bhat, Zerghona Shafia, Rikard Blunck

**Affiliations:** 1 Department of Physics, Université de Montréal, Montréal, QC H3T 1J4, Canada; 2 Department of Pharmacology and Physiology, Université de Montréal, Montréal, QC H3T 1J4, Canada; 3 Interdisciplinary Research Center on Brain and Learning (CIRCA), Université de Montréal, Montréal, QC H3T 1J4, Canada; 4 Department of Neuroscience, Université de Montréal, Montréal, QC H3T 1J4, Canada

**Keywords:** migraine, Kv6 (KCNG), Kv2 (KCNB), genetic variant

## Abstract

Migraines are a common type of headache affecting around 15% of the population. The signalling pathways leading to migraines have not been fully understood, but neuronal voltage-gated ion channels, such as KCNG4, have been linked to this pathology. KCNG4 (Kv6.4) is a silent member of the superfamily of voltage-gated potassium (Kv) channels, which expresses in heterotetramers with members of the KCNB (Kv2) family. The genetic variant Kv6.4-L360P has previously been linked to migraines, but their mode of action remains unknown. Here, we characterized the molecular characteristics of Kv6.4-L360P when co-expressed with Kv2.1. We found that Kv6.4-L360P almost completely abolishes Kv2 currents, and we propose that this mechanism in the trigeminal system, linked to the initiation of migraine, leads to the pathology.

## 1. Introduction

The predominant role of voltage-gated ion channels is the generation of membrane excitability [1]. The flow of ions through the concerted opening of various ion channels with different selectivity leads to the propagation of the action potentials in neurons. Voltage-gated potassium (Kv) channels, specifically, take the role of repolarization of the membrane following the depolarization caused by the sodium channels [2,3,4] as well as adjusting excitability of postsynaptic membranes. Kv channels are tetramers where each monomer consists of six transmembrane helices (S1 through S6) [5,6]. S1–S4 form the voltage sensing domain, whose role is to sense the membrane potential. S5–S6 form the pore domain, which lets ions pass through the membrane.

The superfamily of voltage-gated potassium (Kv) channels is divided into 12 families (Kv1–12) [7]. Each Kv family comprises multiple isoforms, which can form functional homo- and heteromers among the family members. The exceptions are the silent Kv channels Kv5, -6, -8 and -9 [8,9]. The silent Kv channels cannot form functional channels at the plasma membrane by themselves but require co-expression with members of the Kv2 channel family (Kv2.1-2) to form heteromeric channels that have altered gating kinetics compared to Kv2 wildtype channels [10,11,12,13]. In the case of Kv6, these heteromeric channels comprise an alternating subtype (Kv2-6-2-6) [14]. The isoform 4 of Kv6 has been shown to have very little influence on the activation kinetics of co-expressed Kv2.1. However, closed-state inactivation is shifted strongly to more polarized potentials in their presence (see below for details). The role of the silent Kv channels is thus to modulate neuronal excitability by co-expression with non-silent channels. While Kv2.1 is ubiquitously expressed, silent subunits have a more restricted expression [12] which would indicate that their role is more tissue-specific by modulating the properties of the membrane in specific positions to produce the required membrane excitability. In the brain, the silent subunits are more abundantly expressed than Kv2 channels, indicating that most Kv2 channels will be regulated by them. Kv6 channels, specifically, are found throughout the brain but not in the retina.

Lafrenière et al. [15] identified the genetic variant KCNG4-L360P in a patient suffering from migraines. During a migraine, activation of the trigeminal ganglion leads to vasoconstriction via inflammatory neuropeptides [16]. While K_ATP_ and BK_Ca_ channels are involved in vasoconstriction, the role of the Kv channels in the trigeminal ganglion seems more important for the initiation of migraine. There is a wide variety of Kv channels expressed in nociceptors and DRG neurons including Kv1, Kv2, Kv3, Kv4 and Kv7 [17]. They are spatially differentially expressed with the Kv1 channels primarily in the axons and Soma, Kv2 in the axon initial segment and soma, Kv4 in soma and dendrites and Kv3 and Kv7 throughout. The role of the Kv channels is—in addition to repolarizing the membrane after a depolarization—to adjust excitability. The availability of the polarizing K^+^ currents decides the threshold of the depolarizing sodium or calcium currents and thereby also regulates firing frequency.

Here, we characterize the effect of the Kv6.4-L360P variant on the Kv2.1 channel function when co-expressed in Xenopus oocytes. Xenopus oocytes were selected as a model system because we can control the exact amount of mRNA injected and can exclude any effect during transcription. Furthermore, Xenopus oocytes do not express Kv2 channels so we can exclude any influence by endogenous expression.

## 2. Results

**Figure 1:**
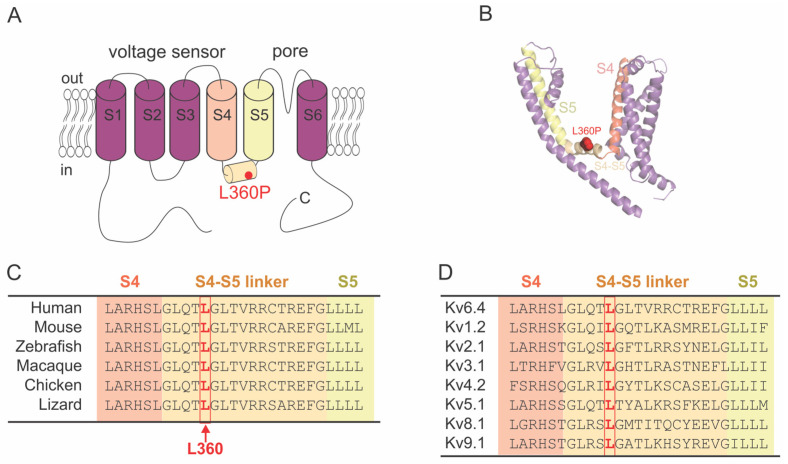
(**A**) Topology of a Kv6.4 monomer. The mutation L360P is located in the S4–S5 linker. (**B**) Structural model of the Kv6.4 monomer generated using Alphafold 3 [18]. N- and C-termini are removed for clarity. (**C**,**D**) Alignment of the amino acids spanning the end of S4 helix, the S4–S5 linker and the beginning of the S6 helix in Kv6.4 across different species (**C**) and in different Kv channels (**D**). The leucine at position 360 in Kv6.4 (highlighted in red) is highly conserved in both instances.

Based on a homology model, L360P is located in the middle of the S4–S5 linker connecting the voltage-sensing domain to the pore domain (Figure 1A,B). The leucine at this position is highly conserved both among different voltage-gated potassium channel families and among different species (Figure 1C,D). The dihedral angles of a proline are not compatible with the alpha-helical secondary structure of the S4–S5 linker. The homology model therefore shows a slight kink in the structure but otherwise superposes with the wildtype structure. This is not surprising as it has been generated by Alphafold 3 without further optimization.

**Figure 2:**
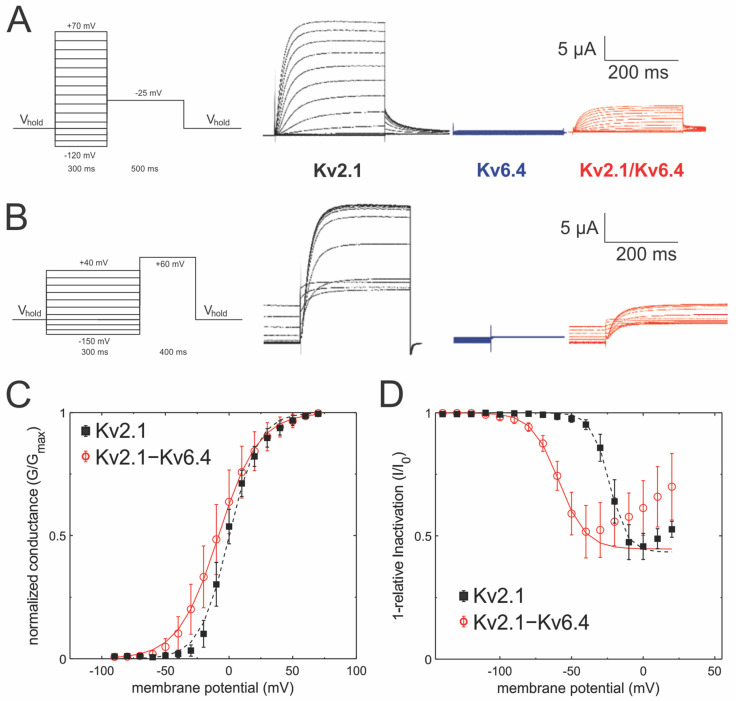
Functional effect of Kv6.4 on Kv2.1. (**A**) Currents elicited from Xenopus oocytes injected with the indicated constructs in response to membrane potential protocol shown on the left. The conductance protocol begins from the holding potential (−120 mV) to testing potentials in the range from −120 mV to +70 mV in increments of 10 mV, followed by a −25 mV to determine instantaneous GV. (**B**) Currents elicited from Xenopus oocytes injected with the indicated constructs in response to membrane potential protocol shown on the left. The inactivation protocol begins at a holding potential (−120 mV) to a range of 4 s long inactivating pulses in the range of −150 mV to +40 mV in increments of 10 mV, followed by a depolarizing pulse to +60 mV in order to measure the level of inactivation caused by the preconditioning pulse (**C**) Conductance–voltage curve for Kv2.1 (N = 8) and Kv2.1/Kv6.4 (N = 18). The curves were fitted with a single Boltzmann equation. (**D**) Inactivation–voltage curve for Kv2.1 (N = 9) Kv2.1/Kv6.4 (N = 11). These curves were fitted with a single reverse Boltzmann equation.

As mentioned above, Kv6.4 channels do not express as homotetramers but have been shown to express in a 2:2 stoichiometry [14]. The Kv2.1/Kv6.4 heteromers show activation and deactivation kinetics very similar to Kv2.1, as reported previously (Figure 2A). This is reflected in the conductance voltage (GV) characteristics (Figure 2C), which is only slightly shifted by ~9 mV and exhibits a shallower slope (Figure 2C). The apparent gating charge is lowered from z_app_ = 2.37 ± 0.14 to z_app_ = 1.63 ± 0.05. We showed previously [14] that this is caused by the activation being governed by the last subunits to activate, suggesting that Kv6.4 activation is likely shifted to more polarized potentials. In contrast, inactivation, governed by the first subunit to inactivate, is strongly shifted to more polarized potentials (V_½_ = −60.7 mV, Figure 2B,D). The maximum inactivation in both cases reaches ~55–60%, given by the ratio of the current of the control pulse before and after the conditioning pulse to different potentials. The apparent gating charge for inactivation is reduced from 4.2 ± 0.2 in Kv2.1 to 3.1 ± 0.1 in Kv2.1/Kv6.4 heteromers.

**Figure 3:**
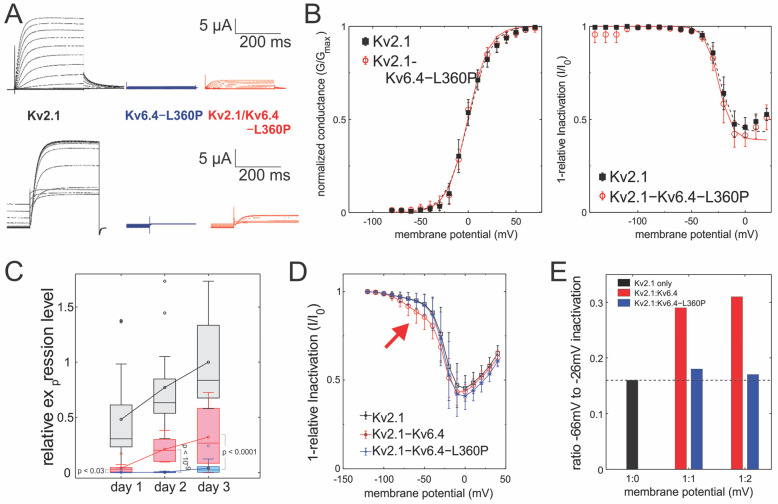
(**A**) Currents elicited from Xenopus oocytes injected with the indicated constructs in response to membrane potential protocols shown in Figure 1A,B. (**B**) Left: conductance–voltage curve for Kv2.1 (N = 8), Kv2.1/Kv6.4 (N = 18) and Kv2.1/Kv6.4-L360P (N = 9). Right: inactivation–voltage curve for Kv2.1 (N = 9), Kv2.1/Kv6.4 (N = 11) and Kv2.1/6.4-L360P (N = 6). All curves were fitted with a single Boltzmann equation. Fitting results are summarized in Table 1. (**C**) Intensity of currents measured was obtained after different incubation times for Kv2.1 (N = 36), Kv2.1/Kv6.4 (N = 31) and Kv2.1/6.4-L360P (N = 50). Boxes indicate upper and lower quartile and median; whiskers indicate the highest and lowest values (outliers are indicated as circles). Line-connected circles indicate mean. (**D**) Conductance–inactivation curves for 1 ng Kv2.1 and variably 1 ng Kv6.4 or Kv6.4-L360P. Arrow emphasizes population of Kv6.4-containing channels. (**E**) Recordings in D in 1:1 and 1:2 ratios were fit to a superposition of two Boltzmann distributions. The fraction of the inactivation curve with *V*_50_ = −66 mV is shown.

To estimate the effect of the migraine-linked genetic variant Kv6.4-L360P, we generated the mutation in Kv6.4-cDNA and in vitro transcribed them before expressing them in Xenopus oocytes, which allowed us to have proper control of the relative expression level of Kv2.1 and Kv6.4 constructs. We expressed Kv6.4-L360P alone and co-expressed it with Kv2.1 in a 10:1 (w 6.4-L360P: w 2.1) ratio. A 10-fold excess of the silent subunit ensures that the expression of the Kv2.1-homotetramers background expression is reduced to a minimum. As expected, no functional expression was observed in the absence of Kv2.1 (Figure 3A). Co-expression with Kv2.1 led to very small currents after three days of expression, compared to one day for Kv6.4 wildtype. There was no significant difference discernable between the currents, kinetics and voltage dependencies compared to the Kv2.1 homotetramers (Figure 3A,B, Table 1). The delayed and low expression and the fact that the characteristics resembled Kv2.1 alone suggests that we are merely recording the Kv2.1 homotetrameric background expression. Accordingly, the currents were small and displayed only after a longer incubation time (18–24 vs. 66–72 h; Table 2).

To ensure that the absence of channels is not due to too slow recovery from inactivation, we tried to apply longer and more hyperpolarized prepulses, which had no effect. While unlikely, it would be possible that the mutation L360P in Kv6.4 removes the strongly shifted inactivation in Kv6.4. It was recently suggested that a leucine in the S6 of Kv2.1 that interacts with another leucine in the S4–S5 linker may be responsible for inactivation in Kv2.1 channels [19]. If this were the case, however, we would not observe a reduction in the expression (Figure 3C).

The increase in homotetrameric Kv2.1 in the presence of Kv6.4-L360P over time, compared to Kv2.1–Kv6.4 heteromers, while also expressing delayed with respect to Kv2.1 homotetramers (Figure 3C) suggests that Kv2.1 is retained longer in the plasma membrane. We next investigated the possibility that Kv6.4-L360P has much stronger expression than Kv6.4 wildtype. In a previous study, we observed that Kv2.1–Kv6.4 complexes in a 3:1 ratio were produced but remained in the ER and were not trafficked to the plasma membrane [14]. We therefore increased the proportion of Kv2.1 in the expression to 1:2 and 1:1 (Kv2:Kv6). For Kv6.4 wildtype, the inactivation characteristics showed two distinct populations following the Kv2.1–Kv6.4 heteromers (*V*_50_ = −66 mV) and the Kv2.1 homotetramers (*V*_50_ = −26 mV; Figure 3D, red arrow). This component accounts for approximately 30% of the inactivation (Figure 3E). For Kv6.4-L360P, however, not even a small fraction of the shifted inactivation curve of Kv2.1–Kv6.4 heteromers was observed. The channels behaved identically to Kv2.1 homotetramers, indicating that Kv6.4-L360P abolishes expression of any functional Kv2–Kv6 complexes.

### 2.1. Kv2.1/Kv6.4-L360P Complexes Are Strongly Translated and Assembled

**Figure 4:**
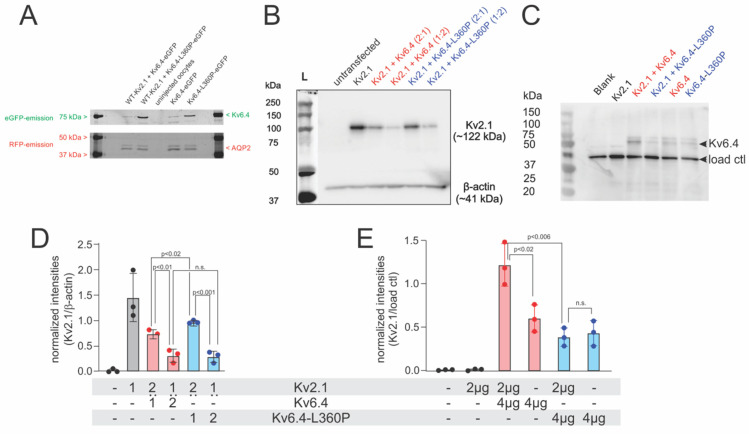
(**A**) SDS page gel from full membranes obtained from Xenopus oocytes. Kv6.4 is fused with eGFP and AQP2 with RFP for detection. eGFP and RFP fluorescence were obtained using appropriate filter sets. The same gels were imaged with both filter sets and superposed here. Aquaporin shows a double band due to glycosylation (N = 3). (**B**) HEK293 cells were transiently transfected with either Kv2.1 (2 µg), Kv2.1:Kv6.4 (2 µg:1 µg), Kv2.1:Kv6.4 (2 µg:4 µg), Kv2.1:Kv6.4-L360P (2 µg:1 µg) or Kv2.1:Kv6.4-L360P (2 µg:4 µg). Untransfected cells were taken as negative controls (first lane). Membrane proteins extracted from these cells were denatured and resolved with SDS-PAGE with anti-Kv2.1 (top) or anti-β-actin (bottom, loading control) antibody. The blot is representative of three independent experiments. (**C**) Plasma membrane expression of Kv6.4 variants after transfection with the indicated DNA determined by the biotinylation assay Western blot with anti-Kv6.4 antibodies, seen as two bands (at ∼57 kDa), observed only in those conditions where Kv6.4 variants were transfected. A non-specific band, owing to low fidelity of the anti-Kv6.4 antibody was observed in all conditions tested. The blot is representative of three independent experiments. (**D**) Densitometric intensities of protein bands of Kv2.1 from B were normalized to those of β-actin in each condition and the resulting ratios were plotted as a box plot that shows mean values ± S.D. (**E**) Densitometric intensities of protein bands of Kv6.4 were normalized to those of the non-specific band in each condition and the resulting ratios were plotted as a box plot that shows mean values + S.D.

Considering that the expression of Kv2.1 is strongly reduced in the presence of the Kv6.4-L360P mutant, the mutation cannot simply lead to a misfolded protein, but Kv2.1–Kv6.4-L360P complexes must be assembled. To verify this, we undertook immunoblotting experiments. We fused Kv6.4 to enhanced GFP (Kv6.4-eGFP) and co-expressed the Kv6.4-eGFP wildtype and -L360P variant with Kv2.1 wildtype as well as on its own. Expression was detected via the eGFP fluorescence from the acrylamide gel. Both Kv6.4 and Kv6.4-L360P were expressed in the absence and presence of Kv2.1 (Figure 4A). In both cases, the expression relative to the Aquaporin 2-RFP control was higher for Kv6.4-L360P, suggesting that the mutant is expressed slightly higher than the wildtype.

We then tested expression in a mammalian expression system; Kv2.1 and Kv6.4-L360P were co-expressed in HEK293 cells (Figure 4A). As expected, Kv2.1 alone is highly expressed, and expression is significantly lowered in the presence of Kv6.4 wildtype (Figure 4B). Increasing the amount of Kv6.4 relative to Kv2.1 further decreased expression. The expression is not lowered, however, in the presence of Kv6.4-L360P compared to Kv6.4-WT, indicating that Kv6.4-L360P is translated and folded properly and forms heterotetramers with Kv2.1. With increasing ratio of Kv2.1 and Kv6.4 or Kv6.4-L360P, the amount of Kv2.1 is significantly decreased. The reduction in the functional expression of Kv2.1/Kv6.4-L360P complexes compared to Kv2.1/Kv6.4 in the electrophysiological data paired with a similar expression in the immunoblotting experiments indicates that (a) Kv6.4-L360P does form tetramers with Kv2.1 and (b) these channels are either not trafficked to the plasma membrane or are not functional.

### 2.2. Kv2.1/Kv6.4-L360P Complexes Are Not Trafficked to the Plasma Membrane

To decide whether the Kv2.1/Kv6.4-L360P is trafficked to the plasma membrane, we carried out a cell-surface biotinylation assay. Kv2.1, Kv2.1/Kv6.4 and Kv2.1/Kv6.4-L360P were expressed in HEK293T cells in a 1:2 molar ratio and plasma membrane proteins exposed to the extracellular solution were biotinylated with sulfo-NHS-esters of biotin. Cells were lysed and the biotinylated proteins were extracted by binding to avidin-coated agarose beads (see methods for details). The oresence of Kv6.4 was observed using the anti-Kv6.4 antibody. The samples with Kv2.1/Kv6.4 showed a robust presence of Kv6.4, whereas Kv2.1 only did not show any presence of Kv6.4 at the plasma membrane (Figure 4C,E). Kv6.4 only showed a faint band. Since it is known that Kv6.4 does not traffic to the surface in the absence of Kv2.1, this band is caused by ER proteins not fully washed off. In the presence of Kv2.1 and the variant Kv6.4-L360P, only the faint background band is observed, indicating that these complexes do not express at the plasma membrane (Figure 4C,E). Since we know that Kv2.1 an Kv6.4-L360P form complexes (Figure 4B,D), these complexes are not trafficked to the surface.

## 3. Discussion

The role of silent Kv channels (KvS) is to modulate Kv2 currents, which is primarily reflected in the voltage dependence of inactivation, shifting its voltage-dependence towards more polarized potentials compared to the Kv2 homomer (Figure 2D). While the Kv2 subunits are more ubiquitously expressed throughout the nervous system, the differential expression patterns of the silent subunits lead to a spatial distribution of the functional phenotypes of the Kv2-KvS complexes. The KvS thus play a regulatory role reducing the availability of Kv2 by (i) lowering expression and (ii) increasing subthreshold inactivation.

The mutation Kv6.4-L360P characterized here is located in the center of the S4–S5 linker. The S4–S5 linker region is essential in Kv for the coupling between the voltage-sensor domain, which responds to changes in the membrane potential, and pore domain, which acts as an effector opening the ion conduction pathway. The coupling is accomplished by annealing to the S6 C-terminus. This link allows for the movement of the S4 to be transmitted to the pore and lets the channel open when the voltage sensors are activated. Mutations in this region in Shaker Kv channels [20] modulate coupling between the voltage sensor and the pore leading to a shifted voltage dependence for the channel, while simultaneously the S4 voltage sensor becomes more sensitive to the membrane potential. The change from a leucine to a proline means a reduction in size and in hydrophobicity and disturbs the alpha-helix forming the S4–S5 linker [21]. Given the position of the mutation, one might suspect that the channels are expressed but not opened due to the disturbed coupling. In this case, however, we should have observed gating currents, as observed for uncoupling mutants in Shaker Kv channels [20], and we should have observed Kv6-L360P channels in the biotinylation assay.

In Kv2.1, the analogous position L316 has been suggested to be involved in the development of inactivation. The mutant L316A led to accelerated inactivation and the inactivation-voltage relation was shifted to more polarized potentials by interaction with the S6 [19]. This would allow for the possibility that the L360P mutation alleviates the strong shift of the Kv6.4-inactivation although it would still be surprising to find the exact value as in Kv2.1. Two other observations argue against this interpretation. First, we observed a strong reduction in functional channels (Figure 2 and Figure 3) despite the fact that Kv2.1 and Kv6.4-L360P are translated as well as Kv2.1 alone (Figure 4), indicating that the assembled channels are either not functional or not trafficked. Secondly, we recently studied 10 different genetic variants in KCNB2 (Kv2.2) linked to neurodevelopmental disease [22]. These genetic variants were spread throughout the channel in the three-dimensional fold but they all still influenced inactivation in KCNB2, indicating that it is not only the coupling region that has a strong influence on the development of inactivation.

Many genetic variants in genes encoding for Kv channels are linked to a variety of neurological and cardiac diseases. The silent Kv, specifically, was linked to cone dystrophy in the eye leading to a loss of sight (Kv8.2 co-expressed with KCNB1) [23] and epilepsy (Kv8) [24,25]. Kv6.4 has not only been linked to migraines in the variant characterized in the present study [15] but also to reduced labor pain when expressed in uterine sensory neurons [26]. We also found in an unrelated study that the family member Kv6.1 is upregulated in airway-innervating nociceptor neurons in a model of allergic airway inflammation [27].

To understand the mechanism of how downregulation of Kv2.1/Kv6.4 leads to a migraine, we have to look at the expression of these channels in the trigeminal ganglion. The major pathway to the development of migraines is the trigemino-vascular path, where excitation of trigeminal neurons triggers vasodilation [28,29]. Both Kv2.1 and Kv6.4 are expressed in the trigeminal ganglion restricted to neuronal cells [30,31,32]. The expression of Kv6.4 is most frequently detected in near-projecting (NP), somatostatin and TRPM8 neurons. TRPM8 has consistently been linked to migraines in genetic studies [33]. Kv6.4 was also found in neurofilaments 1 and 2 and peptidergic neurons but was found here in a lower percentage as Kv2.1.

Postmortem RNA sequencing data from human trigeminal ganglia showed an average ratio of Kv2.1:Kv6.4 of 3:1. Specifically for NP or TRPM8 neurons, this would translate into a ratio of Kv2.1:Kv6.4 = 1:3, indicating that Kv6.4 is expressed in excess. There was no up- or downregulation found in migraine patients versus the control group [34]. The L360P variant would lower the Kv2.1 availability in the NP and TRPM8 neurons, thereby increasing excitability. This would be a similar mechanism as suggested for the migraine-linked variants of the KCNK18 (TRESK) channel [35,36].

The Kv6.4-V419M mutation linked to reduced labor pain [26] showed a very similar phenotype. Also, this mutant was not trafficked to the plasma membrane. However, the authors suggested that Kv2.1 was still reaching the plasma membrane in contrast to our findings (Figure 2A). Absence of Kv6.4 without a reduction in Kv2.1 expression would lead to a reduction in excitability in DRG neurons of the uterus and thus reduce pain, whereas downregulation of Kv2.1 by Kv6.4-L360P increases excitability and causes more pain (migraine).

As a final note, during the review process of this article, another article characterizing the Kv6.4-L360P mutation was posted on a preprint server [37].

## 4. Materials and Methods

### 4.1. Pulse Protocols and Data Analysis

Voltage protocols for the activation and inactivation measurements are shown in Figure 1. Holding potential was adjusted based on the potential at which the measured channels opened. The normalized activation curve was fitted to a Boltzmann equation $\frac{G}{Gmax}=\frac{1}{1+e^{\left(\frac{V_{50}-V}{slope}\right)}}$ where *V*_50_ is the voltage at which 50% of the channels are open or inactivated and *slope* is the slope factor. The apparent charge *z* was defined as $z=\frac{RT}{slope*F}$ where *T* is the temperature in Kelvins (298 K in our case) and *R* is the ideal gas constant 8.314 J mol^−1^ K^−1^. The inactivation was fitted with a reverse Boltzmann equation $Inactivation=\frac{1-Imin}{1+e^{\left(\frac{V_{50}-V}{slope}\right)}}+Imin$ where *V*_50_ is the voltage at which 50% of the channels are open or inactivated, and *slope* is the slope factor. *Imin* is the lowest point of the curve, usually around 0.4 meaning 60% inactivation.

### 4.2. Expression of Kv2/Kv6 in Xenopus Oocytes

Kv2.1 and Kv6.4-eGFP were introduced into the pBSta and pSP64 vectors, respectively, and cRNA was in vitro transcribed by using a T7 mMachine kit (Invitrogen, Waltham, MA, USA), according to the manufacturer’s protocol. The L360P mutation was introduced in Kv6.4-eGFP by PCR mutagenesis and verified by sequencing. Oocytes from *Xenopus laevis* were surgically obtained, according to protocols approved by the Comité de déontologie de l’expérimentation sur les animaux de l’Université de Montréal. Oocytes were injected with mRNA (1) 1 ng Kv2.1, (2) 5 ng Kv2.1 + 50 ng Kv6.4-eGFP and (3) 5 ng Kv2.1 + 50 ng Kv6.4-L360P-eGFP. Injected oocytes were incubated for 24 to 48 h at 18 °C.

### 4.3. Immunoblotting of Kv2.1 and Kv6.4

#### 4.3.1. Xenopus Oocytes

Per condition, 10 injected oocytes were homogenized in 200 µL phosphate-buffered saline (PBS), filled up to 1000 µL and centrifuged 5 min at 180× *g*. The supernatant was collected and centrifuged for 20 min at 20,000× *g*. The pellet was collected and resuspended in 30 µL PBS and 10 µL Laemmli buffer was added. Sixteen µL was loaded on a 10% acrylamide gel.

The samples were injected with mRNA (1) 5 ng Kv2.1 and 50 ng Kv6.4-eGFP, (2) 5 ng Kv2.1 and 50 ng Kv6.4-L360P-eGFP, (3) 50 ng Kv6.4-eGFP, (4) 50 ng Kv6.4-L360P-eGFP as well as (5) uninjected oocytes. Every injected oocyte was co-injected with 5 ng of AQP2-RFP for the loading control. The calibration ladder used was Precision Plus Protein Dual Color Standards (BioRad, Hercules, CA, USA, #1610374). The same gel was imaged with eGFP and RFP filter sets, i.e., blue excitation light and green imaging filter for eGFP and green excitation light and red imaging filter for RFP.

#### 4.3.2. Mammalian Cell Lines

HEK293 cells were seeded in Dulbecco’s modified Eagle’s medium containing 10% fetal calf serum and 1% pencillin/streptomycin on day 1 at a density of 300,000 cells/well in 6-well plates. On day 2, each well was transfected with a fixed concentration of Kv2.1 plasmids (2 µg) and variable concentrations of either WT-Kv6.4 or Kv6.4-L360P (0, 1 or 4 µg) using an in-house polyethylene imine transfection reagent. The total amount of DNA transfected per well was bought up to 6 µg using pcDNA3.1 as a control vector. On day 3, the cells were washed 3 times with ice-cold phosphate-buffered saline and lysed with a buffer containing Tris·HCl, pH 8.0, 150 mm NaCl, 1% dodecyl maltoside, 1 mm EDTA, and protease inhibitors (CompleteTM, Roche Applied Science, Basel, Switzerland). The detergent-insoluble material was subsequently removed by centrifugation (16,000× *g* for 15 min at 4 °C) and the protein concentration of the supernatant was determined. Twenty μg of total proteins per condition were mixed with a sample buffer containing SDS and β-mercaptoethanol, denatured at 45 °C for 30 min, and electrophoretically resolved in denaturing polyacrylamide gels. After transfer of the proteins onto nitrocellulose membranes, the blots were probed with an antibody against either Kv2.1 (rabbit, ab194523, Abcam, Cambridge, UK) or β-actin (mouse, ab49900, Abcam) at recommended dilutions. This was followed by the addition of the horseradish peroxidase (HRP)-conjugated secondary antibody (1:3000) and the ensuing immunoreactivity was detected by chemiluminescence and quantified using ImageJ (ver. 1.54g, https://imagej.net/).

#### 4.3.3. Cell Surface Biotinylation Assay

For cell surface biotinylation experiments, HEK293 cells were seeded and transfected in 6-well plates in the same manner mentioned above. Cells were transfected with either pcDNA3.1 (6 µg), WT-Kv2.1 (2 µg), WT-Kv2.1:WT-Kv6.4 (2 µg:4 µg), WT-Kv2.1:Kv6.4-L360P (2 µg:4 µg), WT-Kv6.4 (4 µg) or Kv6.4-L360P (4 µg). The total amount of DNA to transfect in each condition was brought to 6 µg by adding appropriate amounts of empty pcDNA3.1 vector. The next day, the cells were washed twice with ice-cold PBS followed by incubation with 1 mg/mL of membrane impermeant EZ-Link™ Sulfo-NHS-SS-Biotin (Thermofisher Scientific, Waltham, MA, USA) in PBS for 30 min on ice. This was followed by 3 washes with ice cold quenching solution (50 mM glycine in PBS). The cells were treated with lysis buffer (see above) and the lysates were collected in 1.5 mL microcentrifuge tubes. The tubes were centrifugated at 16,000× *g* for 15 min at 4 °C. The supernatant was separated from the detergent insoluble fraction and quantified. Five-hundred µg of total biotinylated protein per condition was mixed with 50 μL 50% slurry of Pierce™ NeutrAvidin™ Agarose (Thermofisher Scientific) and rotated overnight at 4 °C. The next day, the unbound fractions were separated from the resins by centrifugation at 5000 rpm for 1 min and biotinylated protein bound resins were washed 4 times with lysis buffer. After the final wash, 60 µL of 4× Laemelli buffer was added to the resin and incubated for 30 min at 45 °C for protein denaturation. Twenty-four µL of the sample was loaded and resolved by SDS-PAGE and transferred onto nitrocellulose membranes. The blots were probed by an antibody against Kv6.4 (rabbit, ab155772, Abcam). The rest of the protocol is the same as mentioned above.

## Figures and Tables

**Table 1:** Results from a single Boltzmann fit for the Kv2.1, Kv2.1/Kv6.4 and Kv2.1/Kv6.4-L360P.

**Conductance**	***V*_50_ (mV)**	**Slope (mV)**	**Apparent Charge**		**N**
Kv2.1	0.1 ± 0.8	10.9 ± 0.7	2.37 ± 0.14		6
Kv2.1/Kv6.4	−9.1 ± 0.6	15.9 ± 0.5	1.63 ± 0.05		18
Kv2.1/Kv6.4-L360P	−0.4 ± 0.6	10.7 ± 0.6	2.41 ± 0.12		9
**Inactivation**	***V*** **_50_** **(mV)**	**Slope (mV)**	**Apparent Charge**	**Maximum Inactivation**	**N**
Kv2.1	−23.8 ± 0.4	−6.0 ± 0.3	−4.19 ± 0.21	0.58 ± 0.02	9
Kv2.1/Kv6.4	−60.7 ± 0.6	−8.4 ± 0.4	−3.07 ± 0.14	0.55 ± 0.02	11
Kv2.1/Kv6.4-L360P	−24.7 ± 0.6	−6.1 ± 0.5	−4.24 ± 0.33	0.61 ± 0.02	6

**Table 2:** Level of expressions for Kv2.1 (N = 36), Kv2.1/Kv6.4 (N = 31) and Kv2.1/Kv6.4-L360P (N = 50). The oocytes were injected with 5ng of Kv2.1 and 50ng of Kv6.4 or Kv6.4-L360P depending on the channel studied.

Incubation Time	Constructs	Expression (µA)	Ratio to Kv2.1 (%)	
18–24 h	Kv2.1	33.2 ± 30.5	-	
	Kv2.1/Kv6.4	2.9 ± 3.6	8.7	}*p* < 0.03
	Kv2.1/Kv6.4-L360P	0.2 ± 0.3	0.6	
36–48 h	Kv2.1	53.3 ± 28.5	-	
	Kv2.1/Kv6.4	14.6 ± 6.9	27.4	}*p* < 10^−9^
	Kv2.1/Kv6.4-L360P	0.2 ± 0.3	0.4	
66–72 h	Kv2.1	69.2 ± 28.8	-	
	Kv2.1/Kv6.4	22.3 ± 18.4	32.2	}*p* < 0.0001
	Kv2.1/Kv6.4-L360P	2.9 ± 4.0	4.2	

## Data Availability

All data and materials will be made available upon request.
